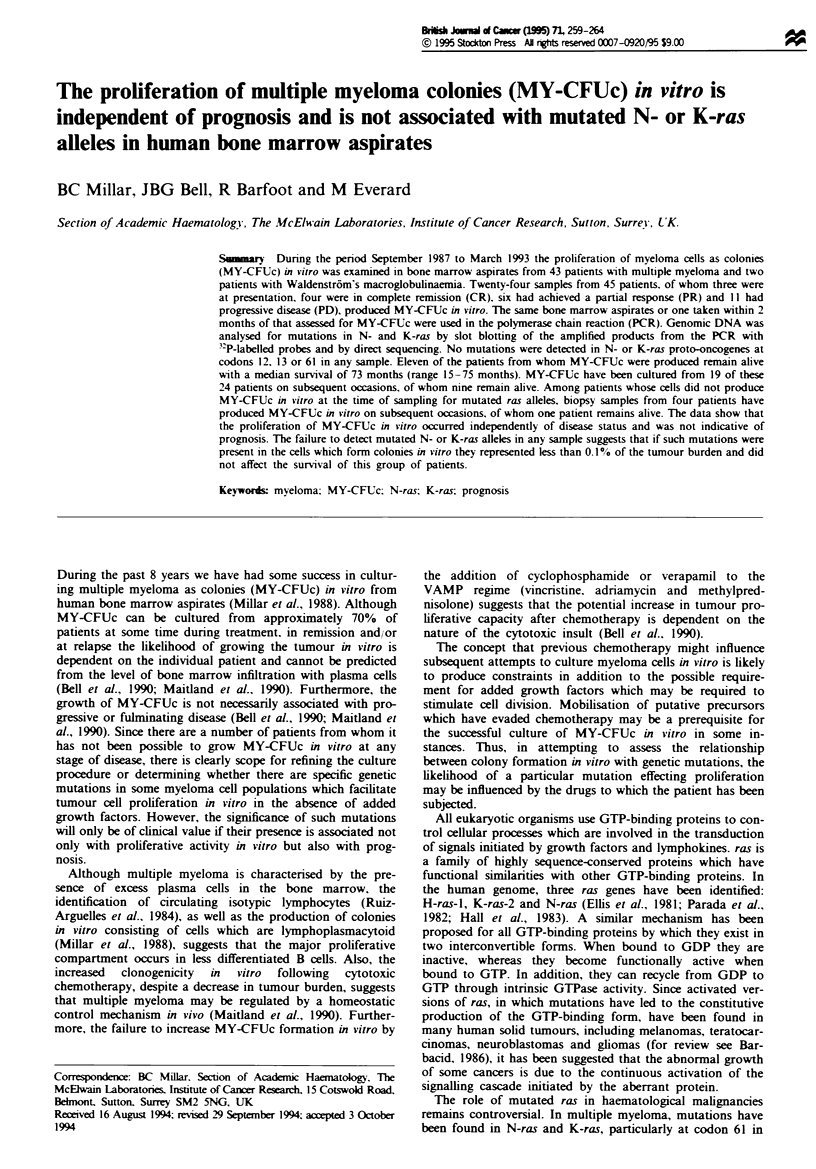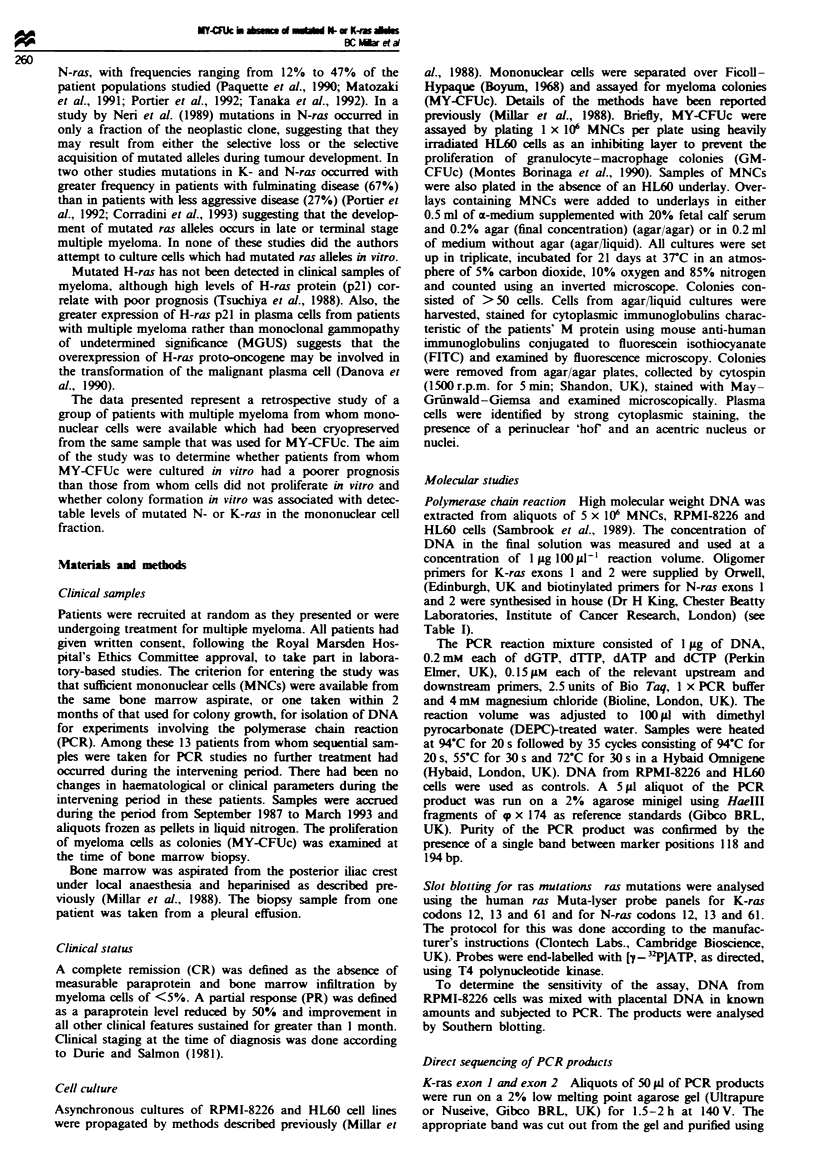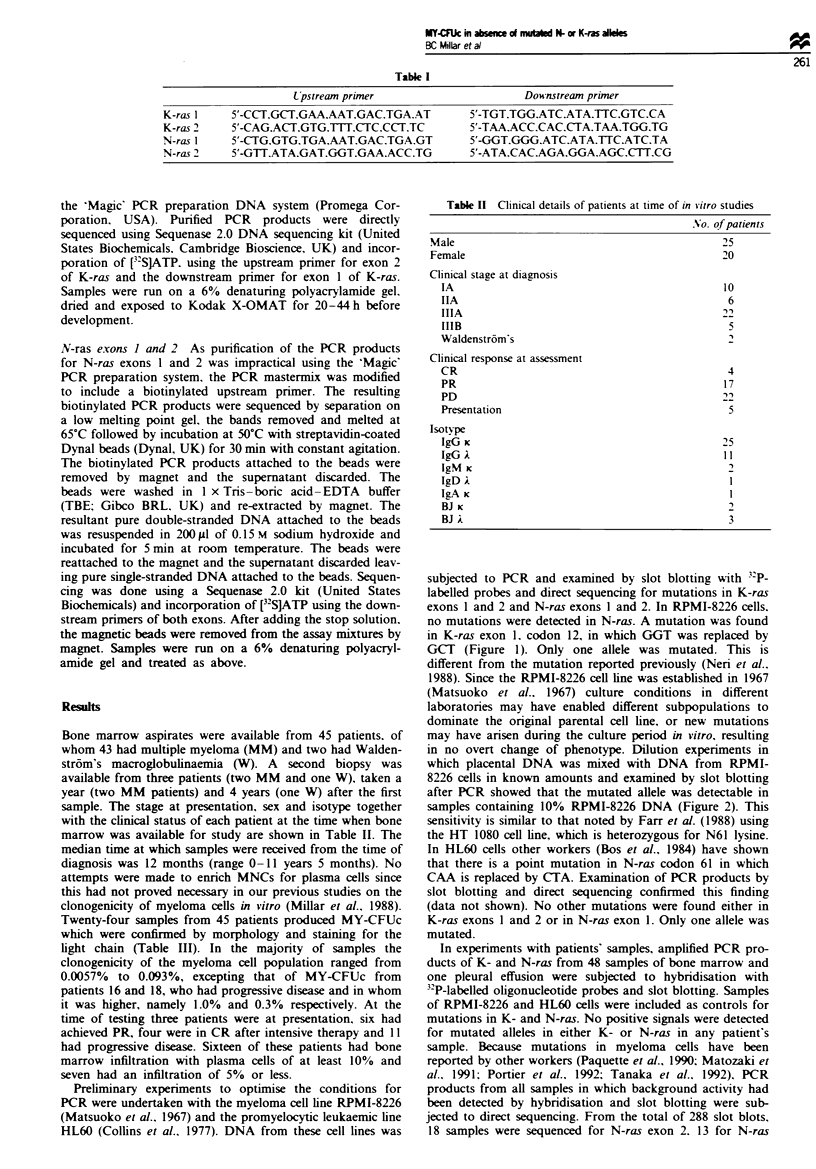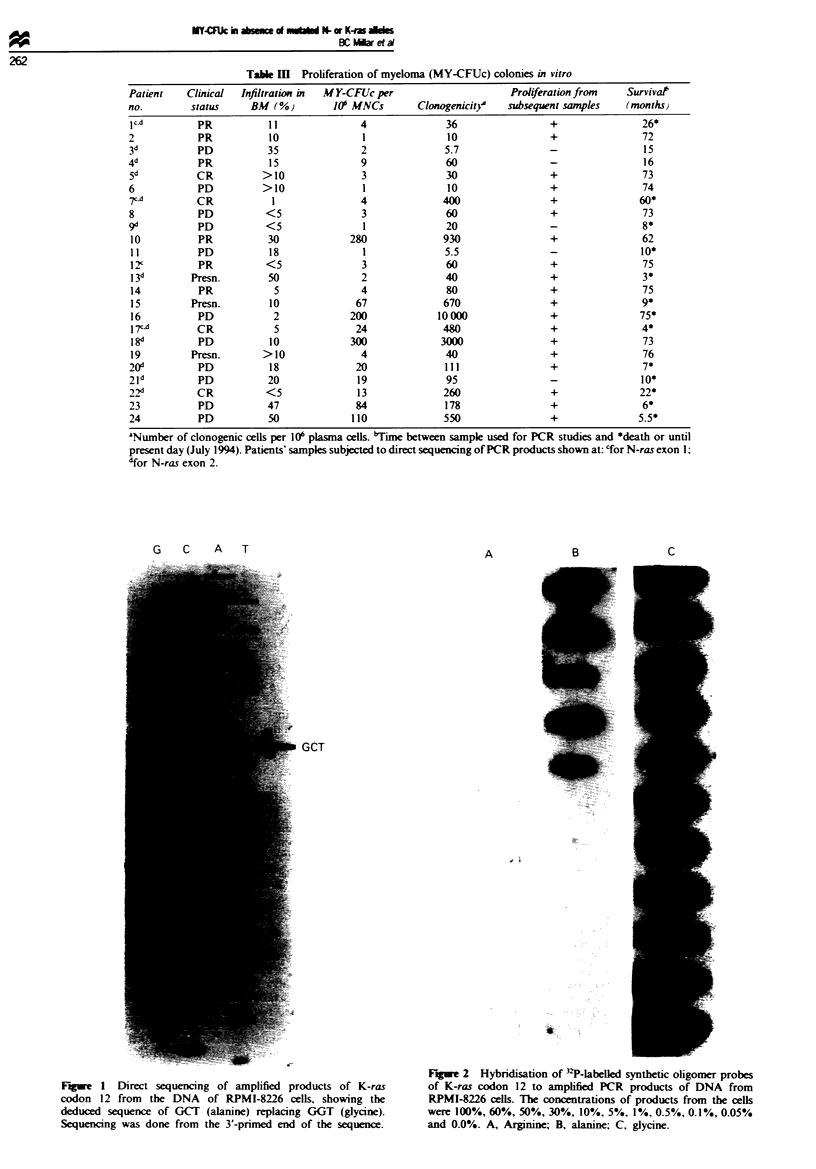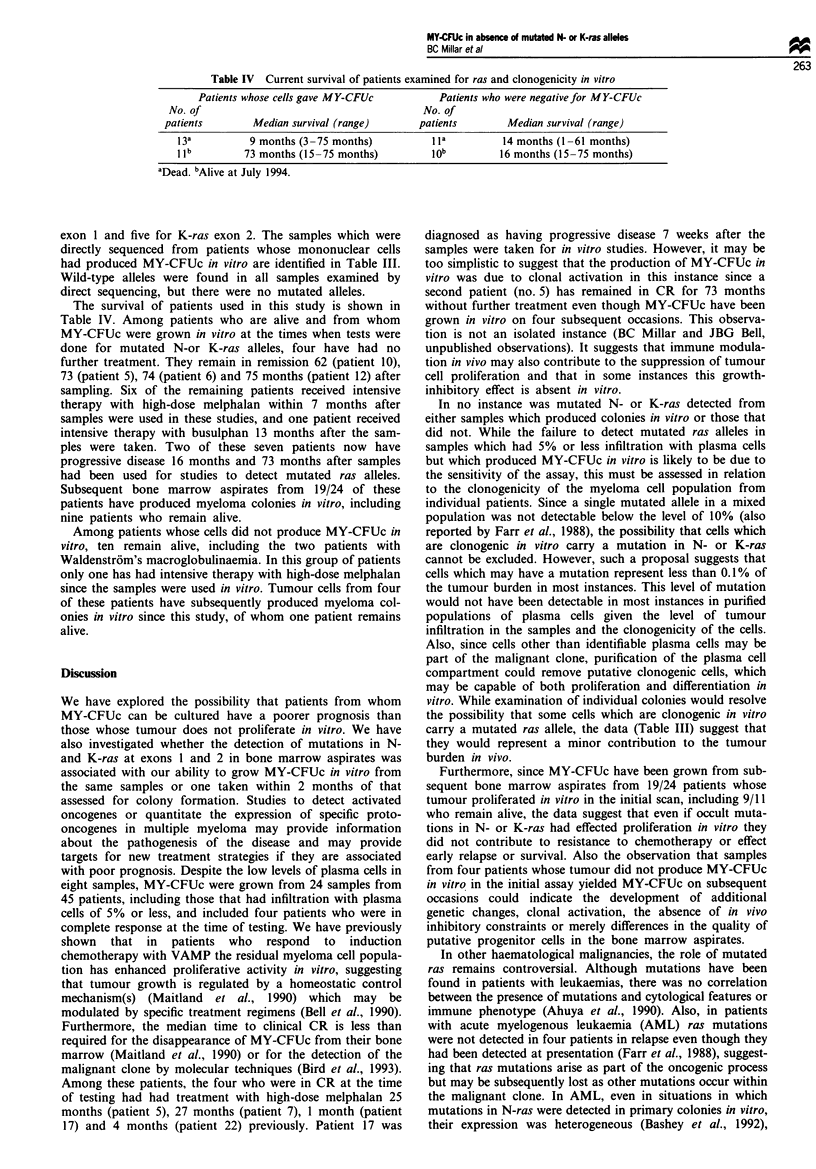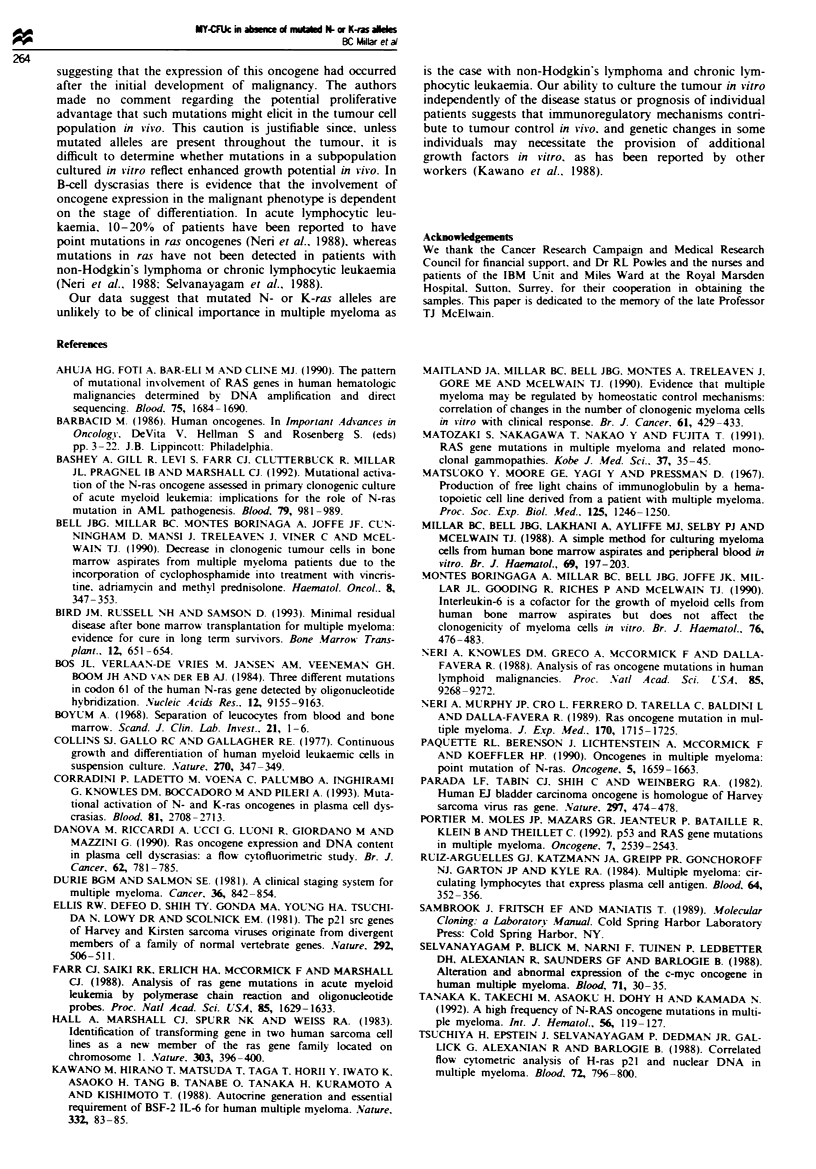# The proliferation of multiple myeloma colonies (MY-CFUc) in vitro is independent of prognosis and is not associated with mutated N- or K-ras alleles in human bone marrow aspirates.

**DOI:** 10.1038/bjc.1995.53

**Published:** 1995-02

**Authors:** B. C. Millar, J. B. Bell, R. Barfoot, M. Everard

**Affiliations:** Section of Academic Haematology, McElwain Laboratories, Institute of Cancer Research, Sutton, Surrey, UK.

## Abstract

**Images:**


© 1995 Stockton Press All rights reserved 0007-0920/95 $9.00

### The proliferation of multiple myeloma colonies (MY-CFUc) *in vitro* is independent of prognosis and is not associated with mutated N- or K-*ras* alleles in human bone marrow aspirates

BC Millar, JBG Bell, R Barfoot and M Everard

*Section of Academic Haematology, The McElwain Laboratories, Institute of Cancer Research, Sutton, Surrey, UK.*

**Summary** During the period September 1987 to March 1993 the proliferation of myeloma cells as colonies (MY-CFUc) *in vitro* was examined in bone marrow aspirates from 43 patients with multiple myeloma and two patients with Waldenström's macroglobulinaemia. Twenty-four samples from 45 patients, of whom three were at presentation, four were in complete remission (CR), six had achieved a partial response (PR) and 11 had progressive disease (PD), produced MY-CFUc *in vitro*. The same bone marrow aspirates or one taken within 2 months of that assessed for MY-CFUc were used in the polymerase chain reaction (PCR). Genomic DNA was analysed for mutations in N- and K-*ras* by slot blotting of the amplified products from the PCR with $^{32}$P-labelled probes and by direct sequencing. No mutations were detected in N- or K-*ras* proto-oncogenes at codons 12, 13 or 61 in any sample. Eleven of the patients from whom MY-CFUc were produced remain alive with a median survival of 73 months (range 15-75 months). MY-CFUc have been cultured from 19 of these 24 patients on subsequent occasions, of whom nine remain alive. Among patients whose cells did not produce MY-CFUc *in vitro* at the time of sampling for mutated *ras* alleles, biopsy samples from four patients have produced MY-CFUc *in vitro* on subsequent occasions, of whom one patient remains alive. The data show that the proliferation of MY-CFUc *in vitro* occurred independently of disease status and was not indicative of prognosis. The failure to detect mutated N- or K-*ras* alleles in any sample suggests that if such mutations were present in the cells which form colonies *in vitro* they represented less than 0.1% of the tumour burden and did not affect the survival of this group of patients.

**Keywords:** myeloma; MY-CFUc; N-*ras*; K-*ras*; prognosis

During the past 8 years we have had some success in culturing multiple myeloma as colonies (MY-CFUc) *in vitro* from human bone marrow aspirates (Millar *et al.*, 1988). Although MY-CFUc can be cultured from approximately 70% of patients at some time during treatment, in remission and or at relapse the likelihood of growing the tumour *in vitro* is dependent on the individual patient and cannot be predicted from the level of bone marrow infiltration with plasma cells (Bell *et al.*, 1990; Maitland *et al.*, 1990). Furthermore, the growth of MY-CFUc is not necessarily associated with progressive or fulminating disease (Bell *et al.*, 1990; Maitland *et al.*, 1990). Since there are a number of patients from whom it has not been possible to grow MY-CFUc *in vitro* at any stage of disease, there is clearly scope for refining the culture procedure or determining whether there are specific genetic mutations in some myeloma cell populations which facilitate tumour cell proliferation *in vitro* in the absence of added growth factors. However, the significance of such mutations will only be of clinical value if their presence is associated not only with proliferative activity *in vitro* but also with prognosis.

Although multiple myeloma is characterised by the presence of excess plasma cells in the bone marrow, the identification of circulating isotypic lymphocytes (Ruiz-Arguelles *et al.*, 1984), as well as the production of colonies *in vitro* consisting of cells which are lymphoplasmacytoid (Millar *et al.*, 1988), suggests that the major proliferative compartment occurs in less differentiated B cells. Also, the increased clonogenicity *in vitro* following cytotoxic chemotherapy, despite a decrease in tumour burden, suggests that multiple myeloma may be regulated by a homeostatic control mechanism *in vivo* (Maitland *et al.*, 1990). Furthermore, the failure to increase MY-CFUc formation *in vitro* by the addition of cyclophosphamide or verapamil to the VAMP regime (vincristine, adriamycin and methylprednisolone) suggests that the potential increase in tumour proliferative capacity after chemotherapy is dependent on the nature of the cytotoxic insult (Bell *et al.*, 1990).

The concept that previous chemotherapy might influence subsequent attempts to culture myeloma cells *in vitro* is likely to produce constraints in addition to the possible requirement for added growth factors which may be required to stimulate cell division. Mobilisation of putative precursors which have evaded chemotherapy may be a prerequisite for the successful culture of MY-CFUc *in vitro* in some instances. Thus, in attempting to assess the relationship between colony formation *in vitro* with genetic mutations, the likelihood of a particular mutation effecting proliferation may be influenced by the drugs to which the patient has been subjected.

All eukaryotic organisms use GTP-binding proteins to control cellular processes which are involved in the transduction of signals initiated by growth factors and lymphokines. *ras* is a family of highly sequence-conserved proteins which have functional similarities with other GTP-binding proteins. In the human genome, three *ras* genes have been identified: H-*ras*-1, K-*ras*-2 and N-*ras* (Ellis *et al.*, 1981; Parada *et al.*, 1982; Hall *et al.*, 1983). A similar mechanism has been proposed for all GTP-binding proteins by which they exist in two interconvertible forms. When bound to GDP they are inactive, whereas they become functionally active when bound to GTP. In addition, they can recycle from GDP to GTP through intrinsic GTPase activity. Since activated versions of *ras*, in which mutations have led to the constitutive production of the GTP-binding form, have been found in many human solid tumours, including melanomas, teratocarcinomas, neuroblastomas and gliomas (for review see Barbacid, 1986), it has been suggested that the abnormal growth of some cancers is due to the continuous activation of the signalling cascade initiated by the aberrant protein.

The role of mutated *ras* in haematological malignancies remains controversial. In multiple myeloma, mutations have been found in N-*ras* and K-*ras*, particularly at codon 61 in

Correspondence: BC Millar, Section of Academic Haematology, The McElwain Laboratories, Institute of Cancer Research, 15 Cotswold Road, Belmont, Sutton, Surrey SM2 5NG, UK
Received 16 August 1994; revised 29 September 1994; accepted 3 October 1994

N-*ras*, with frequencies ranging from 12% to 47% of the patient populations studied (Paquette *et al.*, 1990; Matozaki *et al.*, 1991; Portier *et al.*, 1992; Tanaka *et al.*, 1992). In a study by Neri *et al.* (1989) mutations in N-*ras* occurred in only a fraction of the neoplastic clone, suggesting that they may result from either the selective loss or the selective acquisition of mutated alleles during tumour development. In two other studies mutations in K- and N-*ras* occurred with greater frequency in patients with fulminating disease (67%) than in patients with less aggressive disease (27%) (Portier *et al.*, 1992; Corradini *et al.*, 1993) suggesting that the development of mutated *ras* alleles occurs in late or terminal stage multiple myeloma. In none of these studies did the authors attempt to culture cells which had mutated *ras* alleles *in vitro*.

Mutated H-*ras* has not been detected in clinical samples of myeloma, although high levels of H-*ras* protein (p21) correlate with poor prognosis (Tsuchiya *et al.*, 1988). Also, the greater expression of H-*ras* p21 in plasma cells from patients with multiple myeloma rather than monoclonal gammopathy of undetermined significance (MGUS) suggests that the overexpression of H-*ras* proto-oncogene may be involved in the transformation of the malignant plasma cell (Danova *et al.*, 1990).

The data presented represent a retrospective study of a group of patients with multiple myeloma from whom mononuclear cells were available which had been cryopreserved from the same sample that was used for MY-CFUc. The aim of the study was to determine whether patients from whom MY-CFUc were cultured *in vitro* had a poorer prognosis than those from whom cells did not proliferate *in vitro* and whether colony formation *in vitro* was associated with detectable levels of mutated N- or K-*ras* in the mononuclear cell fraction.

### Materials and methods

### *Clinical samples*

Patients were recruited at random as they presented or were undergoing treatment for multiple myeloma. All patients had given written consent, following the Royal Marsden Hospital's Ethics Committee approval, to take part in laboratory-based studies. The criterion for entering the study was that sufficient mononuclear cells (MNCs) were available from the same bone marrow aspirate, or one taken within 2 months of that used for colony growth, for isolation of DNA for experiments involving the polymerase chain reaction (PCR). Among these 13 patients from whom sequential samples were taken for PCR studies no further treatment had occurred during the intervening period. There had been no changes in haematological or clinical parameters during the intervening period in these patients. Samples were accrued during the period from September 1987 to March 1993 and aliquots frozen as pellets in liquid nitrogen. The proliferation of myeloma cells as colonies (MY-CFUc) was examined at the time of bone marrow biopsy.

Bone marrow was aspirated from the posterior iliac crest under local anaesthesia and heparinised as described previously (Millar *et al.*, 1988). The biopsy sample from one patient was taken from a pleural effusion.

### *Clinical status*

A complete remission (CR) was defined as the absence of measurable paraprotein and bone marrow infiltration by myeloma cells of <5%. A partial response (PR) was defined as a paraprotein level reduced by 50% and improvement in all other clinical features sustained for greater than 1 month. Clinical staging at the time of diagnosis was done according to Durie and Salmon (1981).

### *Cell culture*

Asynchronous cultures of RPMI-8226 and HL60 cell lines were propagated by methods described previously (Millar *et al.*, 1988). Mononuclear cells were separated over Ficoll–Hypaque (Boyum, 1968) and assayed for myeloma colonies (MY-CFUc). Details of the methods have been reported previously (Millar *et al.*, 1988). Briefly, MY-CFUc were assayed by plating $1 \times 10^6$ MNCs per plate using heavily irradiated HL60 cells as an inhibiting layer to prevent the proliferation of granulocyte–macrophage colonies (GM-CFUc) (Montes Borinaga *et al.*, 1990). Samples of MNCs were also plated in the absence of an HL60 underlay. Overlays containing MNCs were added to underlays in either 0.5 ml of α-medium supplemented with 20% fetal calf serum and 0.2% agar (final concentration) (agar/agar) or in 0.2 ml of medium without agar (agar/liquid). All cultures were set up in triplicate, incubated for 21 days at 37°C in an atmosphere of 5% carbon dioxide, 10% oxygen and 85% nitrogen and counted using an inverted microscope. Colonies consisted of >50 cells. Cells from agar/liquid cultures were harvested, stained for cytoplasmic immunoglobulins characteristic of the patients' M protein using mouse anti-human immunoglobulins conjugated to fluorescein isothiocyanate (FITC) and examined by fluorescence microscopy. Colonies were removed from agar/agar plates, collected by cytospin (1500 r.p.m. for 5 min; Shandon, UK), stained with May–Grünwald–Giemsa and examined microscopically. Plasma cells were identified by strong cytoplasmic staining, the presence of a perinuclear 'hof' and an acentric nucleus or nuclei.

### *Molecular studies*

*Polymerase chain reaction* High molecular weight DNA was extracted from aliquots of $5 \times 10^6$ MNCs, RPMI-8226 and HL60 cells (Sambrook *et al.*, 1989). The concentration of DNA in the final solution was measured and used at a concentration of 1 μg 100 $\mu l^{-1}$ reaction volume. Oligomer primers for K-*ras* exons 1 and 2 were supplied by Orwell, (Edinburgh, UK and biotinylated primers for N-*ras* exons 1 and 2 were synthesised in house (Dr H King, Chester Beatty Laboratories, Institute of Cancer Research, London) (see Table I).

The PCR reaction mixture consisted of 1 μg of DNA, 0.2 mM each of dGTP, dTTP, dATP and dCTP (Perkin Elmer, UK), 0.15 μM each of the relevant upstream and downstream primers, 2.5 units of Bio *Taq*, 1 × PCR buffer and 4 mM magnesium chloride (Bioline, London, UK). The reaction volume was adjusted to 100 μl with dimethyl pyrocarbonate (DEPC)-treated water. Samples were heated at 94°C for 20 s followed by 35 cycles consisting of 94°C for 20 s, 55°C for 30 s and 72°C for 30 s in a Hybaid Omnigene (Hybaid, London, UK). DNA from RPMI-8226 and HL60 cells were used as controls. A 5 μl aliquot of the PCR product was run on a 2% agarose minigel using *Hae*III fragments of φ × 174 as reference standards (Gibco BRL, UK). Purity of the PCR product was confirmed by the presence of a single band between marker positions 118 and 194 bp.

*Slot blotting for* ras *mutations* *ras* mutations were analysed using the human *ras* Muta-lyser probe panels for K-*ras* codons 12, 13 and 61 and for N-*ras* codons 12, 13 and 61. The protocol for this was done according to the manufacturer's instructions (Clontech Labs., Cambridge Bioscience, UK). Probes were end-labelled with [$\gamma - ^{32}P$]ATP, as directed, using T4 polynucleotide kinase.

To determine the sensitivity of the assay, DNA from RPMI-8226 cells was mixed with placental DNA in known amounts and subjected to PCR. The products were analysed by Southern blotting.

### *Direct sequencing of PCR products*

*K-*ras *exon 1 and exon 2* Aliquots of 50 μl of PCR products were run on a 2% low melting point agarose gel (Ultrapure or Nuseive, Gibco BRL, UK) for 1.5–2 h at 140 V. The appropriate band was cut out from the gel and purified using

**Table I**

| | Upstream primer | Downstream primer |
|---|---|---|
| K-*ras* 1 | 5′-CCT.GCT.GAA.AAT.GAC.TGA.AT | 5′-TGT.TGG.ATC.ATA.TTC.GTC.CA |
| K-*ras* 2 | 5′-CAG.ACT.GTG.TTT.CTC.CCT.TC | 5′-TAA.ACC.CAC.CTA.TAA.TGG.TG |
| N-*ras* 1 | 5′-CTG.GTG.TGA.AAT.GAC.TGA.GT | 5′-GGT.GGG.ATC.ATA.TTC.ATC.TA |
| N-*ras* 2 | 5′-GTT.ATA.GAT.GGT.GAA.ACC.TG | 5′-ATA.CAC.AGA.GGA.AGC.CTT.CG |

the 'Magic' PCR preparation DNA system (Promega Corporation, USA). Purified PCR products were directly sequenced using Sequenase 2.0 DNA sequencing kit (United States Biochemicals, Cambridge Bioscience, UK) and incorporation of [$^{32}$S]ATP, using the upstream primer for exon 2 of K-*ras* and the downstream primer for exon 1 of K-*ras*. Samples were run on a 6% denaturing polyacrylamide gel, dried and exposed to Kodak X-OMAT for 20–44 h before development.

*N*-ras *exons 1 and 2* As purification of the PCR products for N-*ras* exons 1 and 2 was impractical using the 'Magic' PCR preparation system, the PCR mastermix was modified to include a biotinylated upstream primer. The resulting biotinylated PCR products were sequenced by separation on a low melting point gel, the bands removed and melted at 65°C followed by incubation at 50°C with streptavidin-coated Dynal beads (Dynal, UK) for 30 min with constant agitation. The biotinylated PCR products attached to the beads were removed by magnet and the supernatant discarded. The beads were washed in 1 × Tris–boric acid–EDTA buffer (TBE; Gibco BRL, UK) and re-extracted by magnet. The resultant pure double-stranded DNA attached to the beads was resuspended in 200 μl of 0.15 M sodium hydroxide and incubated for 5 min at room temperature. The beads were reattached to the magnet and the supernatant discarded leaving pure single-stranded DNA attached to the beads. Sequencing was done using a Sequenase 2.0 kit (United States Biochemicals) and incorporation of [$^{32}$S]ATP using the downstream primers of both exons. After adding the stop solution, the magnetic beads were removed from the assay mixtures by magnet. Samples were run on a 6% denaturing polyacrylamide gel and treated as above.

### Results

Bone marrow aspirates were available from 45 patients, of whom 43 had multiple myeloma (MM) and two had Waldenström's macroglobulinaemia (W). A second biopsy was available from three patients (two MM and one W), taken a year (two MM patients) and 4 years (one W) after the first sample. The stage at presentation, sex and isotype together with the clinical status of each patient at the time when bone marrow was available for study are shown in Table II. The median time at which samples were received from the time of diagnosis was 12 months (range 0–11 years 5 months). No attempts were made to enrich MNCs for plasma cells since this had not proved necessary in our previous studies on the clonogenicity of myeloma cells *in vitro* (Millar *et al.*, 1988). Twenty-four samples from 45 patients produced MY-CFUc which were confirmed by morphology and staining for the light chain (Table III). In the majority of samples the clonogenicity of the myeloma cell population ranged from 0.0057% to 0.093%, excepting that of MY-CFUc from patients 16 and 18, who had progressive disease and in whom it was higher, namely 1.0% and 0.3% respectively. At the time of testing three patients were at presentation, six had achieved PR, four were in CR after intensive therapy and 11 had progressive disease. Sixteen of these patients had bone marrow infiltration with plasma cells of at least 10% and seven had an infiltration of 5% or less.

Preliminary experiments to optimise the conditions for PCR were undertaken with the myeloma cell line RPMI-8226 (Matsuoko *et al.*, 1967) and the promyelocytic leukaemic line HL60 (Collins *et al.*, 1977). DNA from these cell lines was

**Table II** Clinical details of patients at time of *in vitro* studies

| | *No. of patients* |
|---|---|
| Male | 25 |
| Female | 20 |
| Clinical stage at diagnosis | |
| IA | 10 |
| IIA | 6 |
| IIIA | 22 |
| IIIB | 5 |
| Waldenström's | 2 |
| Clinical response at assessment | |
| CR | 4 |
| PR | 17 |
| PD | 22 |
| Presentation | 5 |
| Isotype | |
| IgG κ | 25 |
| IgG λ | 11 |
| IgM κ | 2 |
| IgD λ | 1 |
| IgA κ | 1 |
| BJ κ | 2 |
| BJ λ | 3 |

subjected to PCR and examined by slot blotting with $^{32}$P-labelled probes and direct sequencing for mutations in K-*ras* exons 1 and 2 and N-*ras* exons 1 and 2. In RPMI-8226 cells, no mutations were detected in N-*ras*. A mutation was found in K-*ras* exon 1, codon 12, in which GGT was replaced by GCT (Figure 1). Only one allele was mutated. This is different from the mutation reported previously (Neri *et al.*, 1988). Since the RPMI-8226 cell line was established in 1967 (Matsuoko *et al.*, 1967) culture conditions in different laboratories may have enabled different subpopulations to dominate the original parental cell line, or new mutations may have arisen during the culture period *in vitro*, resulting in no overt change of phenotype. Dilution experiments in which placental DNA was mixed with DNA from RPMI-8226 cells in known amounts and examined by slot blotting after PCR showed that the mutated allele was detectable in samples containing 10% RPMI-8226 DNA (Figure 2). This sensitivity is similar to that noted by Farr *et al.* (1988) using the HT 1080 cell line, which is heterozygous for N61 lysine. In HL60 cells other workers (Bos *et al.*, 1984) have shown that there is a point mutation in N-*ras* codon 61 in which CAA is replaced by CTA. Examination of PCR products by slot blotting and direct sequencing confirmed this finding (data not shown). No other mutations were found either in K-*ras* exons 1 and 2 or in N-*ras* exon 1. Only one allele was mutated.

In experiments with patients' samples, amplified PCR products of K- and N-*ras* from 48 samples of bone marrow and one pleural effusion were subjected to hybridisation with $^{32}$P-labelled oligonucleotide probes and slot blotting. Samples of RPMI-8226 and HL60 cells were included as controls for mutations in K- and N-*ras*. No positive signals were detected for mutated alleles in either K- or N-*ras* in any patient's sample. Because mutations in myeloma cells have been reported by other workers (Paquette *et al.*, 1990; Matozaki *et al.*, 1991; Portier *et al.*, 1992; Tanaka *et al.*, 1992), PCR products from all samples in which background activity had been detected by hybridisation and slot blotting were subjected to direct sequencing. From the total of 288 slot blots, 18 samples were sequenced for N-*ras* exon 2, 13 for N-*ras*

**Table III** Proliferation of myeloma (MY-CFUc) colonies *in vitro*

| *Patient no.* | *Clinical status* | *Infiltration in BM (%)* | *MY-CFUc per $10^6$ MNCs* | *Clonogenicity*[a] | *Proliferation from subsequent samples* | *Survival*[b] *(months)* |
|---|---|---|---|---|---|---|
| 1[c,d] | PR | 11 | 4 | 36 | + | 26* |
| 2 | PR | 10 | 1 | 10 | + | 72 |
| 3[d] | PD | 35 | 2 | 5.7 | − | 15 |
| 4[d] | PR | 15 | 9 | 60 | − | 16 |
| 5[d] | CR | >10 | 3 | 30 | + | 73 |
| 6 | PD | >10 | 1 | 10 | + | 74 |
| 7[c,d] | CR | 1 | 4 | 400 | + | 60* |
| 8 | PD | <5 | 3 | 60 | + | 73 |
| 9[d] | PD | <5 | 1 | 20 | − | 8* |
| 10 | PR | 30 | 280 | 930 | + | 62 |
| 11 | PD | 18 | 1 | 5.5 | − | 10* |
| 12[c] | PR | <5 | 3 | 60 | + | 75 |
| 13[d] | Presn. | 50 | 2 | 40 | + | 3* |
| 14 | PR | 5 | 4 | 80 | + | 75 |
| 15 | Presn. | 10 | 67 | 670 | + | 9* |
| 16 | PD | 2 | 200 | 10 000 | + | 75* |
| 17[c,d] | CR | 5 | 24 | 480 | + | 4* |
| 18[d] | PD | 10 | 300 | 3000 | + | 73 |
| 19 | Presn. | >10 | 4 | 40 | + | 76 |
| 20[d] | PD | 18 | 20 | 111 | + | 7* |
| 21[d] | PD | 20 | 19 | 95 | − | 10* |
| 22[d] | CR | <5 | 13 | 260 | + | 22* |
| 23 | PD | 47 | 84 | 178 | + | 6* |
| 24 | PD | 50 | 110 | 550 | + | 5.5* |

[a]Number of clonogenic cells per $10^6$ plasma cells. [b]Time between sample used for PCR studies and *death or until present day (July 1994). Patients' samples subjected to direct sequencing of PCR products shown at: [c]for N-*ras* exon 1; [d]for N-*ras* exon 2.

**Figure 1** Direct sequencing of amplified products of K-*ras* codon 12 from the DNA of RPMI-8226 cells, showing the deduced sequence of GCT (alanine) replacing GGT (glycine). Sequencing was done from the 3′-primed end of the sequence.

**Figure 2** Hybridisation of $^{32}P$-labelled synthetic oligomer probes of K-*ras* codon 12 to amplified PCR products of DNA from RPMI-8226 cells. The concentrations of products from the cells were 100%, 60%, 50%, 30%, 10%, 5%, 1%, 0.5%, 0.1%, 0.05% and 0.0%. A, Arginine; B, alanine; C, glycine.

Table IV Current survival of patients examined for *ras* and clonogenicity *in vitro*

| *Patients whose cells gave MY-CFUc* | | *Patients who were negative for MY-CFUc* | |
|---|---|---|---|
| *No. of patients* | *Median survival (range)* | *No. of patients* | *Median survival (range)* |
| 13[a] | 9 months (3–75 months) | 11[a] | 14 months (1–61 months) |
| 11[b] | 73 months (15–75 months) | 10[b] | 16 months (15–75 months) |

[a]Dead. [b]Alive at July 1994.

exon 1 and five for K-*ras* exon 2. The samples which were directly sequenced from patients whose mononuclear cells had produced MY-CFUc *in vitro* are identified in Table III. Wild-type alleles were found in all samples examined by direct sequencing, but there were no mutated alleles.

The survival of patients used in this study is shown in Table IV. Among patients who are alive and from whom MY-CFUc were grown *in vitro* at the times when tests were done for mutated N-or K-*ras* alleles, four have had no further treatment. They remain in remission 62 (patient 10), 73 (patient 5), 74 (patient 6) and 75 months (patient 12) after sampling. Six of the remaining patients received intensive therapy with high-dose melphalan within 7 months after samples were used in these studies, and one patient received intensive therapy with busulphan 13 months after the samples were taken. Two of these seven patients now have progressive disease 16 months and 73 months after samples had been used for studies to detect mutated *ras* alleles. Subsequent bone marrow aspirates from 19/24 of these patients have produced myeloma colonies *in vitro*, including nine patients who remain alive.

Among patients whose cells did not produce MY-CFUc *in vitro*, ten remain alive, including the two patients with Waldenström's macroglobulinaemia. In this group of patients only one has had intensive therapy with high-dose melphalan since the samples were used *in vitro*. Tumour cells from four of these patients have subsequently produced myeloma colonies *in vitro* since this study, of whom one patient remains alive.

### Discussion

We have explored the possibility that patients from whom MY-CFUc can be cultured have a poorer prognosis than those whose tumour does not proliferate *in vitro*. We have also investigated whether the detection of mutations in N- and K-*ras* at exons 1 and 2 in bone marrow aspirates was associated with our ability to grow MY-CFUc *in vitro* from the same samples or one taken within 2 months of that assessed for colony formation. Studies to detect activated oncogenes or quantitate the expression of specific proto-oncogenes in multiple myeloma may provide information about the pathogenesis of the disease and may provide targets for new treatment strategies if they are associated with poor prognosis. Despite the low levels of plasma cells in eight samples, MY-CFUc were grown from 24 samples from 45 patients, including those that had infiltration with plasma cells of 5% or less, and included four patients who were in complete response at the time of testing. We have previously shown that in patients who respond to induction chemotherapy with VAMP the residual myeloma cell population has enhanced proliferative activity *in vitro*, suggesting that tumour growth is regulated by a homeostatic control mechanism(s) (Maitland *et al.*, 1990) which may be modulated by specific treatment regimens (Bell *et al.*, 1990). Furthermore, the median time to clinical CR is less than required for the disappearance of MY-CFUc from their bone marrow (Maitland *et al.*, 1990) or for the detection of the malignant clone by molecular techniques (Bird *et al.*, 1993). Among these patients, the four who were in CR at the time of testing had had treatment with high-dose melphalan 25 months (patient 5), 27 months (patient 7), 1 month (patient 17) and 4 months (patient 22) previously. Patient 17 was diagnosed as having progressive disease 7 weeks after the samples were taken for *in vitro* studies. However, it may be too simplistic to suggest that the production of MY-CFUc *in vitro* was due to clonal activation in this instance since a second patient (no. 5) has remained in CR for 73 months without further treatment even though MY-CFUc have been grown *in vitro* on four subsequent occasions. This observation is not an isolated instance (BC Millar and JBG Bell, unpublished observations). It suggests that immune modulation *in vivo* may also contribute to the suppression of tumour cell proliferation and that in some instances this growth-inhibitory effect is absent *in vitro*.

In no instance was mutated N- or K-*ras* detected from either samples which produced colonies *in vitro* or those that did not. While the failure to detect mutated *ras* alleles in samples which had 5% or less infiltration with plasma cells but which produced MY-CFUc *in vitro* is likely to be due to the sensitivity of the assay, this must be assessed in relation to the clonogenicity of the myeloma cell population from individual patients. Since a single mutated allele in a mixed population was not detectable below the level of 10% (also reported by Farr *et al.*, 1988), the possibility that cells which are clonogenic *in vitro* carry a mutation in N- or K-*ras* cannot be excluded. However, such a proposal suggests that cells which may have a mutation represent less than 0.1% of the tumour burden in most instances. This level of mutation would not have been detectable in most instances in purified populations of plasma cells given the level of tumour infiltration in the samples and the clonogenicity of the cells. Also, since cells other than identifiable plasma cells may be part of the malignant clone, purification of the plasma cell compartment could remove putative clonogenic cells, which may be capable of both proliferation and differentiation *in vitro*. While examination of individual colonies would resolve the possibility that some cells which are clonogenic *in vitro* carry a mutated *ras* allele, the data (Table III) suggest that they would represent a minor contribution to the tumour burden *in vivo*.

Furthermore, since MY-CFUc have been grown from subsequent bone marrow aspirates from 19/24 patients whose tumour proliferated *in vitro* in the initial scan, including 9/11 who remain alive, the data suggest that even if occult mutations in N- or K-*ras* had effected proliferation *in vitro* they did not contribute to resistance to chemotherapy or effect early relapse or survival. Also the observation that samples from four patients whose tumour did not produce MY-CFUc *in vitro* in the initial assay yielded MY-CFUc on subsequent occasions could indicate the development of additional genetic changes, clonal activation, the absence of *in vivo* inhibitory constraints or merely differences in the quality of putative progenitor cells in the bone marrow aspirates.

In other haematological malignancies, the role of mutated *ras* remains controversial. Although mutations have been found in patients with leukaemias, there was no correlation between the presence of mutations and cytological features or immune phenotype (Ahuya *et al.*, 1990). Also, in patients with acute myelogenous leukaemia (AML) *ras* mutations were not detected in four patients in relapse even though they had been detected at presentation (Farr *et al.*, 1988), suggesting that *ras* mutations arise as part of the oncogenic process but may be subsequently lost as other mutations occur within the malignant clone. In AML, even in situations in which mutations in N-*ras* were detected in primary colonies *in vitro*, their expression was heterogeneous (Bashey *et al.*, 1992), suggesting that the expression of this oncogene had occurred after the initial development of malignancy. The authors made no comment regarding the potential proliferative advantage that such mutations might elicit in the tumour cell population *in vivo*. This caution is justifiable since, unless mutated alleles are present throughout the tumour, it is difficult to determine whether mutations in a subpopulation cultured *in vitro* reflect enhanced growth potential *in vivo*. In B-cell dyscrasias there is evidence that the involvement of oncogene expression in the malignant phenotype is dependent on the stage of differentiation. In acute lymphocytic leukaemia, 10–20% of patients have been reported to have point mutations in *ras* oncogenes (Neri *et al*., 1988), whereas mutations in *ras* have not been detected in patients with non-Hodgkin's lymphoma or chronic lymphocytic leukaemia (Neri *et al*., 1988; Selvanayagam *et al*., 1988).

Our data suggest that mutated N- or K-*ras* alleles are unlikely to be of clinical importance in multiple myeloma as is the case with non-Hodgkin's lymphoma and chronic lymphocytic leukaemia. Our ability to culture the tumour *in vitro* independently of the disease status or prognosis of individual patients suggests that immunoregulatory mechanisms contribute to tumour control *in vivo*, and genetic changes in some individuals may necessitate the provision of additional growth factors *in vitro*, as has been reported by other workers (Kawano *et al*., 1988).

**Acknowledgements**

We thank the Cancer Research Campaign and Medical Research Council for financial support, and Dr RL Powles and the nurses and patients of the IBM Unit and Miles Ward at the Royal Marsden Hospital, Sutton, Surrey, for their cooperation in obtaining the samples. This paper is dedicated to the memory of the late Professor TJ McElwain.

**References**

AHUJA HG, FOTI A, BAR-ELI M AND CLINE MJ. (1990). The pattern of mutational involvement of RAS genes in human hematologic malignancies determined by DNA amplification and direct sequencing. *Blood*, **75**, 1684–1690.

BARBACID M. (1986). Human oncogenes. In *Important Advances in Oncology*, DeVita V, Hellman S and Rosenberg S. (eds) pp. 3–22. J.B. Lippincott: Philadelphia.

BASHEY A, GILL R, LEVI S, FARR CJ, CLUTTERBUCK R, MILLAR JL, PRAGNEL IB AND MARSHALL CJ. (1992). Mutational activation of the N-ras oncogene assessed in primary clonogenic culture of acute myeloid leukemia: implications for the role of N-ras mutation in AML pathogenesis. *Blood*, **79**, 981–989.

BELL JBG, MILLAR BC, MONTES BORINAGA A, JOFFE JF, CUNNINGHAM D, MANSI J, TRELEAVEN J, VINER C AND McELWAIN TJ. (1990). Decrease in clonogenic tumour cells in bone marrow aspirates from multiple myeloma patients due to the incorporation of cyclophosphamide into treatment with vincristine, adriamycin and methyl prednisolone. *Haematol. Oncol.*, **8**, 347–353.

BIRD JM, RUSSELL NH AND SAMSON D. (1993). Minimal residual disease after bone marrow transplantation for multiple myeloma: evidence for cure in long term survivors. *Bone Marrow Transplant.*, **12**, 651–654.

BOS JL, VERLAAN-DE VRIES M, JANSEN AM, VEENEMAN GH, BOOM JH AND VAN DER EB AJ. (1984). Three different mutations in codon 61 of the human N-ras gene detected by oligonucleotide hybridization. *Nucleic Acids Res.*, **12**, 9155–9163.

BOYUM A. (1968). Separation of leucocytes from blood and bone marrow. *Scand. J. Clin. Lab. Invest.*, **21**, 1–6.

COLLINS SJ, GALLO RC AND GALLAGHER RE. (1977). Continuous growth and differentiation of human myeloid leukaemic cells in suspension culture. *Nature*, **270**, 347–349.

CORRADINI P, LADETTO M, VOENA C, PALUMBO A, INGHIRAMI G, KNOWLES DM, BOCCADORO M AND PILERI A. (1993). Mutational activation of N- and K-ras oncogenes in plasma cell dyscrasias. *Blood*, **81**, 2708–2713.

DANOVA M, RICCARDI A, UCCI G, LUONI R, GIORDANO M AND MAZZINI G. (1990). Ras oncogene expression and DNA content in plasma cell dyscrasias: a flow cytofluorimetric study. *Br. J. Cancer*, **62**, 781–785.

DURIE BGM AND SALMON SE. (1981). A clinical staging system for multiple myeloma. *Cancer*, **36**, 842–854.

ELLIS RW, DEFEO D, SHIH TY, GONDA MA, YOUNG HA, TSUCHIDA N, LOWY DR AND SCOLNICK EM. (1981). The p21 src genes of Harvey and Kirsten sarcoma viruses originate from divergent members of a family of normal vertebrate genes. *Nature*, **292**, 506–511.

FARR CJ, SAIKI RK, ERLICH HA, McCORMICK F AND MARSHALL CJ. (1988). Analysis of ras gene mutations in acute myeloid leukemia by polymerase chain reaction and oligonucleotide probes. *Proc. Natl Acad. Sci. USA*, **85**, 1629–1633.

HALL A, MARSHALL CJ, SPURR NK AND WEISS RA. (1983). Identification of transforming gene in two human sarcoma cell lines as a new member of the ras gene family located on chromosome 1. *Nature*, **303**, 396–400.

KAWANO M, HIRANO T, MATSUDA T, TAGA T, HORII Y, IWATO K, ASAOKO H, TANG B, TANABE O, TANAKA H, KURAMOTO A AND KISHIMOTO T. (1988). Autocrine generation and essential requirement of BSF-2 IL-6 for human multiple myeloma. *Nature*, **332**, 83–85.

MAITLAND JA, MILLAR BC, BELL JBG, MONTES A, TRELEAVEN J, GORE ME AND McELWAIN TJ. (1990). Evidence that multiple myeloma may be regulated by homeostatic control mechanisms: correlation of changes in the number of clonogenic myeloma cells *in vitro* with clinical response. *Br. J. Cancer*, **61**, 429–433.

MATOZAKI S, NAKAGAWA T, NAKAO Y AND FUJITA T. (1991). RAS gene mutations in multiple myeloma and related monoclonal gammopathies. *Kobe J. Med. Sci.*, **37**, 35–45.

MATSUOKO Y, MOORE GE, YAGI Y AND PRESSMAN D. (1967). Production of free light chains of immunoglobulin by a hematopoietic cell line derived from a patient with multiple myeloma. *Proc. Soc. Exp. Biol. Med.*, **125**, 1246–1250.

MILLAR BC, BELL JBG, LAKHANI A, AYLIFFE MJ, SELBY PJ AND McELWAIN TJ. (1988). A simple method for culturing myeloma cells from human bone marrow aspirates and peripheral blood *in vitro*. *Br. J. Haematol.*, **69**, 197–203.

MONTES BORINGAGA A, MILLAR BC, BELL JBG, JOFFE JK, MILLAR JL, GOODING R, RICHES P AND McELWAIN TJ. (1990). Interleukin-6 is a cofactor for the growth of myeloid cells from human bone marrow aspirates but does not affect the clonogenicity of myeloma cells *in vitro*. *Br. J. Haematol.*, **76**, 476–483.

NERI A, KNOWLES DM, GRECO A, McCORMICK F AND DALLA-FAVERA R. (1988). Analysis of ras oncogene mutations in human lymphoid malignancies. *Proc. Natl Acad. Sci. USA*, **85**, 9268–9272.

NERI A, MURPHY JP, CRO L, FERRERO D, TARELLA C, BALDINI L AND DALLA-FAVERA R. (1989). Ras oncogene mutation in multiple myeloma. *J. Exp. Med.*, **170**, 1715–1725.

PAQUETTE RL, BERENSON J, LICHTENSTEIN A, McCORMICK F AND KOEFFLER HP. (1990). Oncogenes in multiple myeloma: point mutation of N-ras. *Oncogene*, **5**, 1659–1663.

PARADA LF, TABIN CJ, SHIH C AND WEINBERG RA. (1982). Human EJ bladder carcinoma oncogene is homologue of Harvey sarcoma virus ras gene. *Nature*, **297**, 474–478.

PORTIER M, MOLES JP, MAZARS GR, JEANTEUR P, BATAILLE R, KLEIN B AND THEILLET C. (1992). p53 and RAS gene mutations in multiple myeloma. *Oncogene*, **7**, 2539–2543.

RUIZ-ARGUELLES GJ, KATZMANN JA, GREIPP PR, GONCHOROFF NJ, GARTON JP AND KYLE RA. (1984). Multiple myeloma: circulating lymphocytes that express plasma cell antigen. *Blood*, **64**, 352–356.

SAMBROOK J, FRITSCH EF AND MANIATIS T. (1989). *Molecular Cloning: a Laboratory Manual*. Cold Spring Harbor Laboratory Press: Cold Spring Harbor, NY.

SELVANAYAGAM P, BLICK M, NARNI F, TUINEN P, LEDBETTER DH, ALEXANIAN R, SAUNDERS GF AND BARLOGIE B. (1988). Alteration and abnormal expression of the c-myc oncogene in human multiple myeloma. *Blood*, **71**, 30–35.

TANAKA K, TAKECHI M, ASAOKU H, DOHY H AND KAMADA N. (1992). A high frequency of N-RAS oncogene mutations in multiple myeloma. *Int. J. Hematol.*, **56**, 119–127.

TSUCHIYA H, EPSTEIN J, SELVANAYAGAM P, DEDMAN JR, GALLICK G, ALEXANIAN R AND BARLOGIE B. (1988). Correlated flow cytometric analysis of H-ras p21 and nuclear DNA in multiple myeloma. *Blood*, **72**, 796–800.